# Surface Activation of Polylactic Acid-Based Wood-Plastic Composite by Atmospheric Pressure Plasma Treatment

**DOI:** 10.3390/ma13204673

**Published:** 2020-10-20

**Authors:** Philipp Sauerbier, Robert Köhler, Gerrit Renner, Holger Militz

**Affiliations:** 1 Wood Biology and Wood Products, Faculty of Forest Sciences, University of Goettingen, Büsgenweg 4, 37077 Göttingen, Germany; holz@uni-goettingen.de; 2 Laboratory of Laser and Plasma Technologies, University of Applied Sciences and Arts, Von-Ossietzky-Str. 99, 37085 Göttingen, Germany; robert.koehler@hawk.de; 3 Instrumental Analytical Chemistry, Faculty of Chemistry, University of Duisburg-Essen, Universitätsstr, 45141 Essen, Germany; gerrit.renner@uni-due.de

**Keywords:** wood-polymer composites, plasma treatment, coatings, polylactic acid

## Abstract

Wood-plastic composite (WPC) based on a polylactic acid (PLA) matrix is a promising material since it is biobased, degradable, sustainable, and 3D printable. However, due to its coloring, visible layers after 3D-printing, and small build volumes of these printers, a coating or gluing of parts might be required. This study investigates the influence of a dielectric barrier discharge (DBD) plasma treatment of PLA-based WPC to activate the surface and improve, e.g., coating capabilities. X-ray photoelectron spectroscopy (XPS) measurements showed the oxidation of the surface due to the formation of carbonyl and carboxyl groups. Laser scanning microscopy revealed a surface roughening after the treatment. Contact angles of water and diiodomethane decreased significantly after the plasma treatment and the consecutively calculated surface free energy increased. Finally, two practical adhesion tests revealed an improvement of the applied acrylic dispersion coating’s adhesion to the WPC surface: The assigned cross-cut class improved, and the pull-off strength increased from 1.4 to 2.3 N/mm^2^.

## 1. Introduction

Wood-plastic composites (WPCs) are a promising material with a growing market that is expected to expand further, driven by increasing attention and demand for environmentally friendly and sustainable products [1]. Incorporating wood fibers and particles provides ecological and economic benefits, as it substitutes parts of the polymer with cheaper, CO_2_-storing, and renewably wood. Life cycle assessment (LCA) revealed the ecological superiority of natural fibers compared to glass fibers [2], while Feifel et al. showed with an LCA that WPC flooring is a tenable ecological alternative to tropical wood even at the first use. Furthermore, the environmental impact can be significantly reduced by material recycling [3]. Studies by Bütün et al. and Krause et al. showed that recovered fibers from fiberboards are a suitable material source for the WPC production. Utilizing fibers from medium-density fiberboards revealed beneficial effects on the produced WPC’s physicomechanical properties [4,5]. However, the change of the physicomechanical properties of a particle or fiber-containing polymer is not only affected by its source, but also significantly depending on the particle size and filling level [6].

While the WPC market in Europe by volume is dominated by polypropylene (PP)-based WPC due to the broad market for floorings and deckings [7], polylactic acid (PLA) WPC is a promising and rising material. It is biobased, biodegradable, and binds CO_2_ during its production as it is made of renewable resources. The referenced review articles provide a comprehensive insight into its production from renewable resources, chemical and physical properties and resulting fields of application [8,9,10]. Additionally, its thermal properties make it an exceptional material for fused deposition modeling (FDM) 3D printing. For this reason, it is already the most common printing material for FDM printers [11]. PLA-based WPC naturally has a yellowish/ocher appearance, which is colorable during the extrusion process but not brilliant, which might require a coating to meet aesthetic requirements. Proper coating adhesion is also desired for 3D printing; the additive manufacturing process leaves visually and haptically individual layers that require filling, sanding, and, finally, coating for a smooth paint finish. Additionally, due to the often very limited maximum print volume of 3D printers, assembling multiple parts is common. Both the coating and the gluing require excellent adhesion.

Various approaches to improve this adhesion exist, e.g., sanding, pretreatment with an adhesion promoter, and plasma treatment have been shown to increase the adhesion of a polyester-based coating on various WPC substrates [12]. Low-temperature plasma treatments are an industry standard nowadays for functionalizing and activating polymer surfaces, especially for alkenes. Benefits include good adhesion after the rapid and chemical-free treatment [13]. The effects of low-temperature plasma treatment’s influence on wood and wood-based products have been investigated more recently. Altgen et al. and Wascher et al. showed that the physicomechanical properties of plywood and fiberboards improved after a plasma treatment of the base wood material [14,15]. Investigations of plasma treatments of injection-molded and/or extruded alkene-based WPCs by Oporto et al. and Hünnekens et al. revealed a better wettability and additionally Hünnekens et al. showed a recombination of the surface and therefore a time-dependent reduction in the surface activation effect [16,17].

This study investigates the impact of a low-temperature plasma treatment at ambient pressure on a commercial PLA-based WPC with 60 % wood content. X-ray photoelectron spectroscopy (XPS) and Fourier-transform infrared spectroscopy (FTIR) are used to investigate changes in the surface chemistry, and laser scanning microscopy (LSM) is utilized to measure changes in the surface morphology. The surface free energy is calculated with determined water and diiodomethane contact angles. Finally, the adhesion of a commercial acrylic dispersion coating is examined.

With this study setup, the authors want to contribute to a better understanding of the modes of action of plasma treatment of PLA-based WPC and improve practical application scenarios. Specifically, for a 3D-printing application, the surface activation provides improved adhesion of a final coating or gluing for part assembly.

## 2. Materials and Methods

### 2.1. Specimen Preparation

A commercially available product with 60% wood, 37% polylactic acid (PLA) polymer, and 3% additives was used to perform the experiments: WPC BIO PLA H60-500-14 (JELU-WERK J. Ehrler GmbH & Co KG, Rosenberg, Germany) [18]. Test specimens were produced by injection molding with an ALLROUNDER 420 C (Arburg, Loßburg, Germany). The heating cylinders’ temperature was set to 155, 165, 175, and 185 °C, with a total cycle time of 43 s.

Specimens were cut into pieces of (100 × 50 × 4) mm^3^ for the pull-off test and (50 × 50 × 4) mm^3^ for all other analysis methods. The samples were stored in normal climate (20 °C, 65% relative humidity) to ensure standardized conditioning. Additionally, all samples, including the untreated references, were wiped with 2-propanol and a lint-free cloth prior to the treatment. 2-propanol was used to remove possible dust, fingerprints, and residues from the injection molding process since it is a widely used cleaning agent. Additionally, it evaporates completely and does not affect the PLA surface.

### 2.2. Plasma Treatment

Plasma treatment of the specimens was performed using a dielectric barrier discharge (DBD) setup. Specimens were placed on 4-mm-thick glass, insulating them from the aluminum ground electrode underneath. The upper electrode consists of a bronze powder filled alumina (Al_2_O_3_) dielectric. A 3-axis cross table was used to statically position the upper electrode (145 × 85 mm^2^) to completely cover the samples (50 × 50 mm^2^ or 100 × 50 mm^2^) with a 2 mm distance. A 150 L/min gas flow of synthetic air (80% nitrogen/20% oxygen) was established inside the gap. This airflow provides a defined working gas for the plasma discharge as well as a cooling for the specimens.

**Figure A1:**
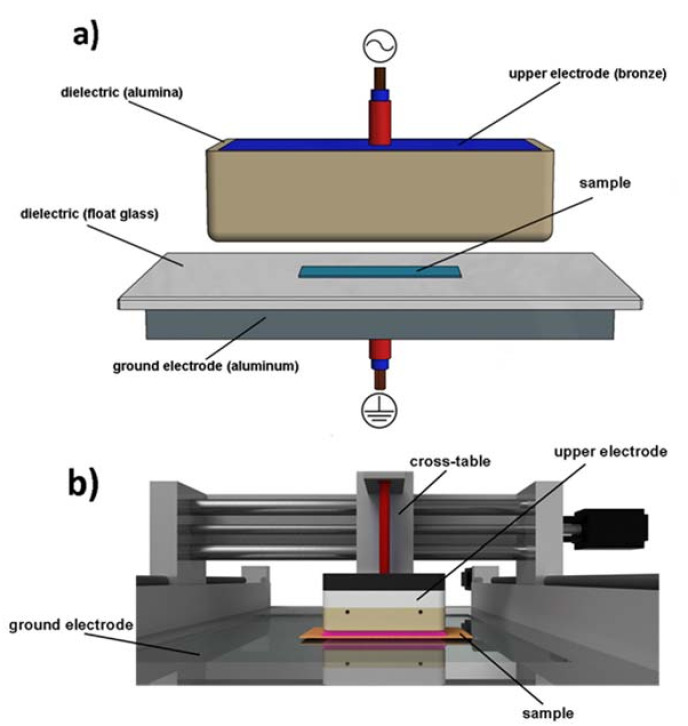
(**a**) Electrode configuration. The sample is placed on a glass-insulated aluminum ground electrode. Above the specimen and a 2 mm treatment gap is the upper electrode (**b**) experimental setup scheme. The upper electrode is mounted on a 3-axis cross table and can be moved over the specimen to ensure an accurate 2 mm treatment gap and complete coverage of the sample with the upper electrode. (Adapted from ten Bosch et al. [19]).

An applied peak-to-peak voltage of approx. 24 kV and a repetition frequency of 17 kHz were used for the plasma treatment with a pulsed sine waveform and a pulsed duration of approx. 1.9 µs. Treatment time was set to 60 s in which 1 s of treatment is followed by 1 s of non-treatment, resulting in a 30 s net treatment. This procedure ensures that the specimen’s surface temperature stays below 60 °C, as shown in a previous study utilizing thermographic analysis on the same plasma device [19]. Schematics of the electrode and the general experimental setup can be found in Figure A1 in the Appendix A.

### 2.3. Analysis

A list of the used analysis equipment with range and accuracies can be found in Table A1 in the Appendix A.

#### 2.3.1. Fourier-Transform Infrared Spectroscopy (FTIR)

FTIR measurements of five samples of each variant were performed on a PerkinElmer Frontier (PerkinElmer LAS (Deutschland) GmbH, Rodgau Jügesheim, Germany). The measurement was performed with diamond ATR (Specac Golden Gate GS 10515, Specac Ltd., Orpington, UK) in a range of 700–4000 cm^−1^, averaged over 64 scans with a resolution of 4 cm^−1^. After a baseline correction, the data were normalized on the maximum peak at 1752 cm^−1^ for the reference and 1753 cm^−1^ for the plasma-treated samples.

#### 2.3.2. X-ray Photoelectron Spectroscopy (XPS)

Measurements were performed with a PHI 5000 Versa Probe II (ULVAC-PHI, Chigasaki, Japan) with monochromatic Al–K_α_ radiation (1486.6 eV of photon energy). The detector’s minimum resolution measured at the Ag3d 5/2 peak was 0.6 eV. Detail-spectra of carbon 1s (C1s), oxygen 1s (O1s) and nitrogen 1s (N1s) with a pass energy of 46.95 eV, a step size of 0.1 eV, and a spot size of 200 µm were collected for three samples. Neutralization of sample charging was carried out to avoid charging effects. The structures were fitted applying Voigt profiles after conducting a Shirley baseline subtraction.

#### 2.3.3. Contact Angle

A Krüss G10 (Krüss GmbH, Hamburg, Germany) was used to determine the contact angle of an apolar (diiodomethane) and a polar liquid (water) on the sample’s surface with the sessile drop technique. Droplets with a volume of 7 µL for diiodomethane and 10 µL for water were dosed, and the resulting angles analyzed using the corresponding software DSA 1 1.90 (Krüss GmbH, Hamburg, Germany) with the Tangent 1 fit, one second after surface contact of the droplets. This procedure ensures that a changing contact angle due to, e.g., the weight of the droplets itself or penetration of the liquids into the specimen, does not affect the results or at least affects all measurements in a reproducible way. Following the approach of Owens, Wendt, Rabel, and Kaelbe (OWRK), the surface free energy, separated in a polar and a dispersive component, can be calculated from the obtained data [20]. The same software was used to calculate the surface free energy and its error.

For each variant, four samples were analyzed with 12 droplets of each liquid per sample.

#### 2.3.4. Laser Scanning Microscopy (LSM)

The surface roughness was measured with a KEYENCE VK-X100 (KEYENCE Deutschland GmbH, Neu-Isenburg, Germany) with a 100× objective.

Five samples were analyzed by measuring twelve (3 × 4) 135 × 101 µm adjacent images. The images were stitched together to the size of approx. 400 × 400 µm and ten squares in the size of 20 × 20 µm^2^ were randomly placed in this area. With the resulting data, the developed interfacial area ratio (Sdr) was calculated according to DIN EN ISO 25178 (2012) [21] without adding any additional filter. To ensure conclusive results and reliable surface data, the specimens were measured at the same position before and after plasma treatment.

#### 2.3.5. Paint Adhesion/Pull-Off Strength/Cross-Cut Test

Paint adhesion of a waterborne acrylic dispersion was tested by applying Alpina Weißlack für Innen (Alpina Farben GmbH, Ober Ramstadt, Germany) with a roll paint. Two layers of coating were applied, each on top of the former after 24 h of drying. The first layer was applied within one hour after plasma treatment. This resulted in a paint load of approx. 80 g/m^2^.

Using these painted specimens, the pull-off strength was determined by the dolly test based on ASTM D4541-02 (2002) [22] and DIN EN ISO 4624 (2016) [23]. In total, fifteen dollies with a 20 mm diameter, three each (100 × 50 × 4) mm^3^ specimen, were glued on the coating’s surface with Araldite 2011 (Huntsman Advanced Materials LLC, Salt Lake City, UT, USA). The glued dollies’ extraction was performed with a hydraulic hand-held measuring device PosiTest AT-P (DeFelsko Corporation, Ogdensburg, NY, USA).

Additionally, a cross-cut test was performed on the painted specimens, according to DIN EN ISO 2409 (2013) [24]. Three samples were cross-cut at three different locations. Since the coating’s thickness was determined to be <60 µm with a digital microscope (KEYENCE VHX-5000 KEYENCE Deutschland GmbH, Neu-Isenburg, Germany), a 1 mm grid was used for the cuts, as required by the standard.

#### 2.3.6. Statistical Analysis

A normal distribution of the results was ensured by a Kolmogorov–Smirnov normality test (α = 0.05). Further statistical analysis utilized a two-sample unequal variance (heteroscedastic) *t*-Test and/or a Tukey’s HSD (honest significant difference) test, both with α = 0.05.

For visualization purposes, boxplots are used. The box contains 50 % of the data points, each whisker 25%. The mean value is represented by a small square, the median by a solid line, and outliers by x.

## 3. Results

In the following, the results of the measurements are presented unbiased. An interpretation will be covered in the discussion.

### 3.1. Fourier-Transform Infrared Spectroscopy (FTIR)

**Figure 1:**
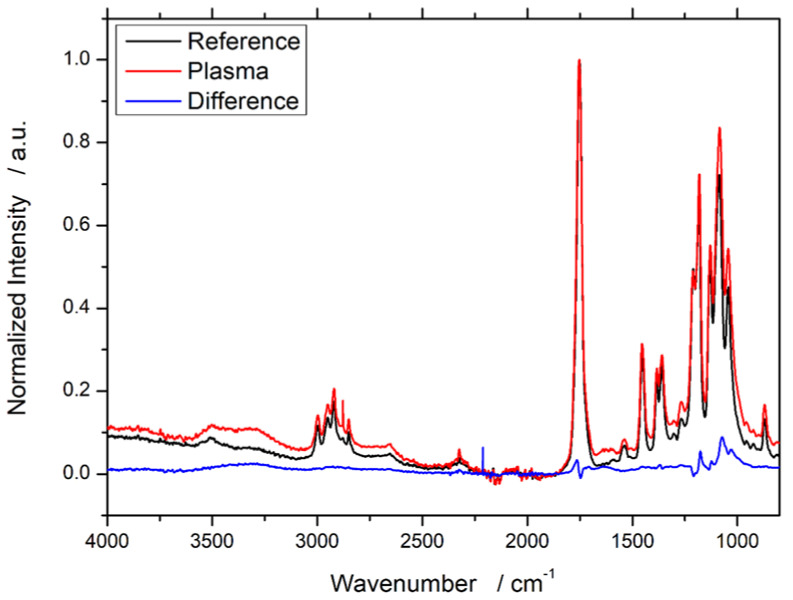
FTIR spectra of WPC with 60% wood content in PLA before and after plasma treatment. Measured spectra are normalized on the max peak (reference = 1752 cm^−1^; plasma-treated = 1753 cm^−1^), and a difference spectrum is calculated (blue line).

The FTIR spectroscopy data are visualized in Figure 1. The obtained spectra before and after plasma treatment are comparable. No new peaks are visible after the treatment. Therefore, a difference plot was calculated and visualized as a blue line to help recognizing changes in the two spectra. Even with this difference spectrum, the changes remain to be minor. Slight changes can only be found around 1000–1250 cm^−1^ (C–C/H) and 1750 cm^−1^ (C=O).

### 3.2. X-ray Photoelectron Spectroscopy (XPS)

**Figure 2:**
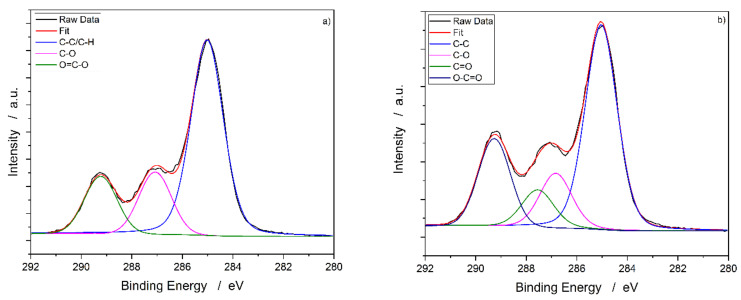
XPS raw data of the C1s carbon peak and fitted Voigt profiles of PLA WPC measurements. (**a**) Spectra before plasma treatment with three fits corresponding to the three bonds present in PLA (C–C/H, C–O and O–C=O). (**b**) The spectra after the treatment reveals an increase in the O=C–O and the formation of a new C=O peak.

The XPS measurement allows for analyzing the elemental composition of a specimen’s surface. Unlike the FTIR data, the XPS-obtained data showed significant changes. Figure 2 shows the C1s peak of a PLA60 specimen before and after the plasma treatment. The spectra were shifted to the main peak of C1s at 285.0 eV [25]. In literature, PLA samples without wood content are described by three structures in C1s peak, attributed to C–C, C–O and O–C=O [26].

Due to the plasma treatment, new functional groups may occur at the substrate surface [27]. Therefore, a split of the carbon peak into four structures is proposed for the plasma-treated samples to cover the main oxygen-containing functional groups: hydroxyl, carbonyl, and carboxyl. The prominent peak at 285.0 eV can be assigned to C–C and C–H. The second peak at around 286.8 eV can be attributed to C–O. The structures at around 287.6 and 289.2 eV can be assigned to C=O and O–C=O [28].

C–C/C–H and C–O concentrations on the surface are reduced due to the plasma treatment, and C=O and O–C=O are formed. The results are presented in Table 1. It has to be noted that while no nitrogen was present in the untreated reference, after plasma treatment, 1 at% in total was detected. Due to the measurement principle, the N– and C-containing groups are expected to overlap in the spectra [29], making it very challenging and non-reliable to fit these peaks—especially given the minor content of only 1 at%. Therefore, the fits will only be done with oxygen functional groups. However, one has to bear in mind that a minor proportion of the plasma-treated C–O, C=O, and O–C=O concentrations contain nitrogen.

### 3.3. Contact Angle

**Figure 3:**
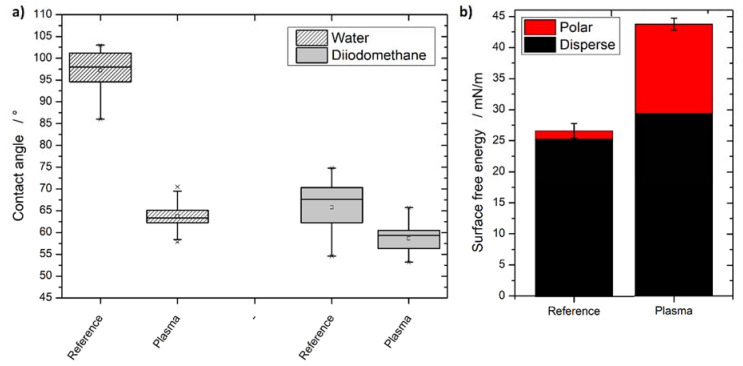
(**a**) Raw contact angle data of water and diiodomethane on PLA60. The contact angles get reduced significantly due to the plasma treatment for both liquids. (**b**) The calculated surface free energy (OWRK) from the obtained contact angles increases after the plasma treatment significantly.

Raw data of the contact angle measurement of both liquids (water and diiodomethane of the PLA specimens before and after plasma treatment) can be found in Figure 3a.

The plasma treatment significantly reduced the water contact angle of the WPC specimens by approx. 35–40°. Initial contact angles of water (97.2 ± 4.6°) and diiodomethane (65.7 ± 5.6) were reduced significantly by the plasma treatment to 63.8 ± 2.7°, respectively, 58.64 ± 3.1°. According to the OWRK approach, this reduction leads to an increase in surface free energy. The calculated results are visualized in Figure 3b and reveal a significant increase in the surface free energy from 26.6 ± 1.2 mN/m to 43.8 ± 0.9 mN/m. Even though the polar part’s growth dominates the general increase in the surface free energy, the dispersive component also contributed to the general increase in the surface free energy.

### 3.4. Laser Scanning Microscopy (LSM)

**Figure 4:**
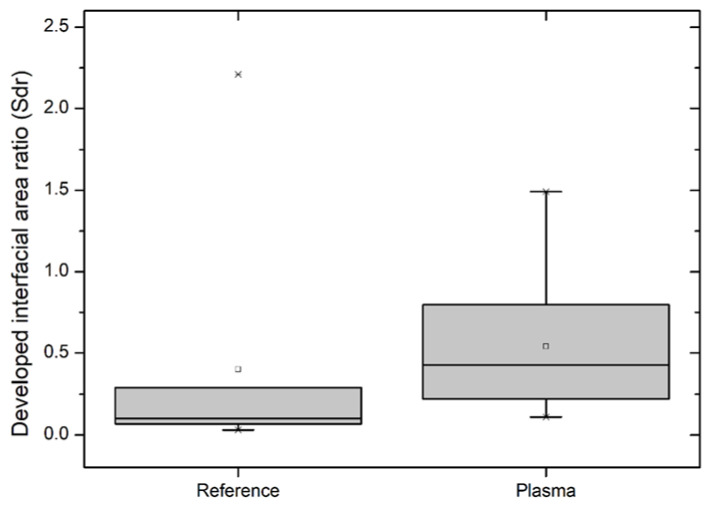
Developed interfacial area ratio (Sdr) of references and plasma-treated PLA WPC specimens calculated according to DIN EN ISO 25178 (2012) [21].

The developed interfacial area ratio (Sdr) was calculated according to DIN EN ISO 25178 (2012) [21]—visualization of the results can be found in Figure 4.

The Sdr value (developed interfacial area ratio) describes the additional area due to the roughness/morphology of a specimen’s surface in relation to its base planar area. A completely flat surface would have an Sdr value of 0. A significant increase in the Sdr from (mean) 0.40 ± 0.6 to 0.54 ± 0.4—resulting in a 35% increase in the mean Sdr or a 10 % increase in the total surface area—results from the plasma treatment. As illustrated by the larger box and the longer whiskers, the plasma treatment also leads to more disperse results. This scatter of the measured values is due to the chaotic and unpredictable nature of plasma treatment on a microscopic level.

### 3.5. Paint Adhesion/Pull-Off Strength/Cross-Cut Test

DIN EN ISO 2409 (2013) [24] (cross-cut test) allows for the classification of a coating’s adhesion on a scale from 0 to 5. Class 5 means that the coating was removed entirely from the surface, while 0 means that the coating is still completely intact, and the cross-cut grid structure remains unaltered. The untreated reference specimens were assigned a mean class of 4.3. After the plasma treatment, the coating showed a better adhesion on the cut-in grid, which resulted in an assigned mean class of 0.2.

**Figure 5:**
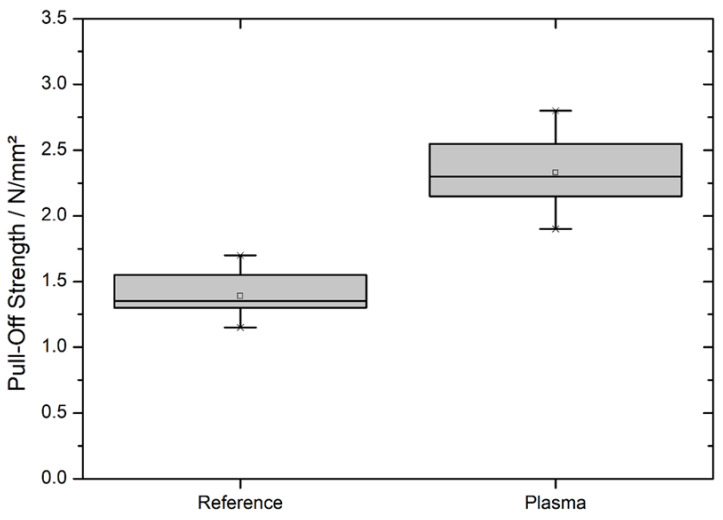
Pull-off strength of a waterborne acrylic dispersion on PLA WPC measured according to ASTM D4541-02 (2002) [22] and DIN EN ISO 4624 (2016) [23] before and after plasma treatment.

For the determination of the pull-off strength according to ASTM D4541-02 (2002) [22] and DIN EN ISO 4624 (2016) [23] of the applied acrylic dispersion, circular-shaped dollies were glued on the coating, pulled off, and the required strength was quantified. The results are shown in Figure 5. It is evident that the pull-off strength significantly increased from 1.4 ± 0.2 to 2.3 ± 0.2 N/mm^2^—corresponding to an increase of 0.9 N/mm^2^ or approx. 67.6%.

## 4. Discussion

The FTIR spectra showed no evaluable changes. Only due to the difference spectrum of the FTIR measurements, minor changes of the FTIR results before and after the treatment are visible. There are various reasons that the FTIR measurement did not show a major and meaningful effect of the plasma treatment, even though all other analysis methods revealed a significant impact. First of all, FTIR results are not easily and always quantifiable; even with a large sample size and profound statistical analysis, a quantification is discussed controversially. Only newly formed peaks are of clear and indisputable meaning. Since no new peaks were generated in the spectra but already existing ones altered, newly generated groups might not be visible. This assumption is substantiated by the fact that the used infrared radiation is supposed to have a penetration depth of approx. 2 µm at 1000 cm^−1^ [30]. The plasma treatment affects only the top layers of the specimen’s surface, approx. in the range of tens of angstrom (10^−10^ m) [31]. Since the penetration depth of the analysis device is three orders of magnitude higher than the treatment depth, it is comprehensible that these changes are not visible in the spectra primarily because they do not provide new peaks.

In contrast, the XPS data show significant changes in the surface’s chemistry. There are two main reasons why XPS is better suited to analyze surface chemistry changes: First of all, a functional group delivers a specific peak in the spectrum. For instance, a carboxyl group (O=C–O(H)) is split in the FTIR spectra showing a signal for carbonyl (C=O) around 1750 cm^−1^ and hydroxyl (C–O(H)) a broad peak from 3200 to 3700 cm^−1^. For the analysis of XPS measurement, the different functional groups are attributed to dedicated peaks. Therefore, contrary to FTIR, newly formed carbonyl groups cannot ’hide’ inside already dominantly present carboxyl groups and are detected as a new functional group. More importantly, the average depth of analysis is approx. 5 nm [32], which is in the same order of magnitude as the stated tens of angstrom of the changes due to the plasma treatment. This maximizes the possibility of analyzing plasma effects and not losing information content due to too high or too low penetration depths of the analysis method.

XPS measurements revealed oxidation of the specimen’s surface. The percentage of unoxidized C–C/C–H as well as the lower oxidation state C–O was reduced, and higher oxidized functional groups, mainly carbonyl but also carboxyl, increased. The formation of new oxygen-containing functional groups and further oxidation of existing ones are the expected DBD plasma treatment outcome with (synthetic) air. However, the amount and the exact type of newly formed oxygen-containing functional groups on PLA are not fixed; they are heavily dependent on the plasma device, exact treatment parameters, and especially the treatment time as previous studies from Vergne et al. on thin PLA films show [33]. Additionally, the results are in agreement with previous findings with these exact plasma parameters and device on polypropylene-based WPC in which it could be shown that predominantly carbonyl groups were generated due to the plasma treatment [34]. With the PLA’s methyl group offering a good position for the described oxidation process and the decrease in C-C/C-H bonds in the XPS spectra, carbonyl groups’ generation seems plausible.

LSM measurements show that the specimen’s surface was roughened, which led to an increase in the total surface area of approx. 10%. In general, an increase in the surface roughness for the utilized plasma treatment is expected. This is in agreement with the following LSM results as well as the theoretically expected ones: the amorphous parts of the polymer matrix are more prone to the plasma etching than the crystalline ones, thus leading to a roughened surface due to the crystalline PLA structures’ higher structural integrity [35].

At first glance, the initial contact angle seems too high but is in good agreement with the literature. Paragkumar et al. showed that PLA’s water contact angle depends heavily on the applied droplets’ dwell time. The initial contact angle is reported to be 96° but decreases to 82° after 60 s. Paragkumar et al. name the dominantly present methyl groups of the PLA on the specimen’s surface as a reason. Due to the applied water, the methyl groups/PLA reorientate, and, therefore, the contact angle is lowered over time [36].

Contact angles for the performed measurements in this study were measured 1 s after the droplet was applied to the surface. This was done to eliminate a potential penetration of the applied liquid into the possible permeable WPC. This not only explains the high measured contact angle but is also in agreement with the surface functionalization shown with XPS. The measured reduction in C–C/C–H and the increase in C=O are heavily dependent on available methyl groups for this reaction. The plasma treatment lowered the contact angle significantly for both liquids—especially for water.

This is the expected outcome since the newly generated functional groups on the surface add a permanent dipole to which hydrogen bonds of water can be formed. This increases the wettability and consequently lowers the contact angle. However, one has to bear in mind that the contact angle is also affected by the surface morphology [37]. The higher surface roughness after plasma treatment will also lower the contact angle for both liquids. Since the contact angle is below 90°, the now wetting surface results in a further reduced contact angle due to the surface chemical changes after plasma treatment. For a non-wetting surface (contact angle >90°), the contact angle is further increased by a rougher surface; however, this only applies for reference’s water contact angle. Therefore, the increase in the calculated surface free energy is mostly, but not solely, attributable to changes in the surface chemistry.

Finally, the adhesion tests offer the possibility to show the combined effects of the chemical and microscopic morphological changes of the PLA surface by a macroscopic and applied experiment. The dolly test revealed that the pull-off strength was increased by about two-thirds of the initial value. A coating starts to be useable in a practical approach when its pull-off strength exceeds 1 N/mm^2^. However, a better adhesion is desired as it offers more resistance against mechanical damage to the coating. Additionally, the acrylic dispersion bonds to the specimen via non-covalent hydrogen bonding—the same way as most glue’s mode of action available on the market—which makes this acrylic dispersion a good benchmark to also predict the improvement of the strength of an applied glue [38]. Furthermore, the water contact angle reduction below 90° changed the surface from non-wetting to wetting. Since the applied acrylic dispersion is waterborne, this is crucial for the coating’s distribution on the surface. A non-wetting surface may result in an air-containing interface reducing its adhesion capability.

The cross-cut test also revealed an improvement in the coating’s adhesion. Together with the dolly test results, this high highlights how the functionalization of the surface with oxygen groups and the higher surface area benefit the coating and, presumably, the gluing capability.

## 5. Conclusions

This study shows a surface modification of a PLA-based WPC with 60% wood content by a dielectric barrier discharge plasma treatment at ambient pressure. Surface chemistry analysis revealed a functionalization/oxidizing. The formation of new carbonyl groups could be shown as well as a percentage increase in the already present carboxyl and hydroxyl groups. Additionally, the surface morphology has been changed, and, as a result, the surface roughness increased. These microscopic and chemical changes, due to the plasma treatment, also led to a change regarding the wettability, as the contact angle measurements of water and diiodomethane revealed. Accordingly, the calculated surface free energy increased—mainly due to an increase in the polar part, which is in agreement with the higher oxidized surface.

Following a more practical approach, the coating adhesion investigation was carried out and revealed the impact of these microscopic, chemical, and surface free energetic alterations on the adhesion of a conventional and commercially available acrylic dispersion coating. Two standardized tests showed an increase in the coating’s adhesion after the plasma treatment. This improvement can help to enable the utilization of coatings for PLA-WPC and addresses its yellowish coloring. However, it is of special interest for 3D printing/additive manufacturing for which an after-treatment of the surface by a filler/coating combination and gluing of small parts is of crucial importance.

## Figures and Tables

**Table 1:** XPS data of the C1s carbon peak from fitted Voigt profiles of PLA WPC before and after plasma treatment.

Specimen	C–C/C–H/at%	C–O/at%	C=O/at%	O–C=O/at%
Reference	64.5	18.5	-	17
Plasma treated	50.4	16.2	10	23.4

## Appendix A

**Table A1:** Equipment list of analytical devices used for the presented study with range and accuracies.

Device	Measured Quantity	Range	Accuracy
Perkin Elmer Frontier	5	8300–350 cm^−1^	0.4 cm^−1^
PHI 5000 VersaProbe II	3	-	0.6 eV
Krüss G10	10	0–180°	0.1°
KEYENCE VK-X100	5	Area (100× magnification lens) 135 × 101 µm^2^	Height (z-axis): 20 nm Width (x&y-axis): 30 nm
PosiTest AT-P	3	0–12 N/mm^2^	0.1 N/mm^2^
